# Highly efficient dual-channel doublet emission in lanthanide cerium(III) complex

**DOI:** 10.1038/s41377-026-02377-4

**Published:** 2026-08-07

**Authors:** Yujia Li, Peiyu Fang, Zeyou Xiong, Jiayin Zheng, Huanyu Liu, Rubing Bai, Zuqiang Bian, Zhiwei Liu

**Affiliations:** Beijing National Laboratory for Molecular Sciences, College of Chemistry and Molecular Engineering, Peking University, Beijing, China

**Keywords:** Optical materials and structures, Optical physics

## Abstract

Dual-channel luminescent materials are attractive due to their unique electronic structures and potential applications in various fields. Different from traditional dual-channel emitters with closed-shell structure showing singlet fluorescence and/or triplet phosphorescence, we demonstrate herein dual-channel cerium(III) complexes with open-shell structure showing doublet 5d-4f emission from the central Ce(III) ion. By systematically designing and studying a series of cerium(III) complexes Ce(Tp^4Me^)(Bp^4Me^)_2_, Ce(Tp^4Me^)_2_(Bp^4Me^) and Ce(Tp^4R^)_3_ (R = Me, Cl, I) with gradually varied coordination numbers from seven to eight and nine, respectively, it is found that the nine-coordinated complexes Ce(Tp^4R^)_3_ showed crowded coordination environments and longer coordination bonds, which underwent a coordination structural relaxation after high energy light excitation, resulting in a new channel emission. This work demonstrates an intriguing dual-channel doublet emission phenomenon in lanthanide Ce(III) complexes and will inspire new insights both into the design of novel lanthanide-based luminescent materials and dual-channel luminescent materials.

## Graphical abstract

**Figure:**
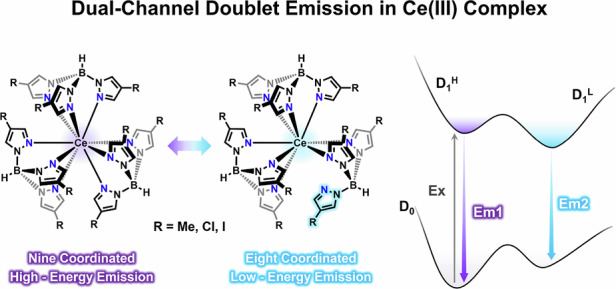

## Introduction

Dual-channel or even multi-channel emission materials have attracted sustained interest owing to their unique electronic structures and potential applications in various fields^1^, such as sensing^2^, multilevel encryption and optical data storage^3–8^, imaging^9–12^, and electroluminescence^13–15^. To date, dual-channel emission has typically been realized through several representative strategies, such as anti-Kasha molecular design that enables emission from higher-lying excited states^16,17^, molecular engineering approach based on isomerization control^18,19^, excited-state population balancing^20–23^, the integration of dual emissive building blocks^24,25^, excimer formation^26–28^, and so on. The deliberate design and systematic investigation of dual-channel emitters enhance our understanding of photophysical processes and broaden the applications of luminescent materials. So far, the reported dual-channel emitters are mainly realized in closed-shell molecules, while open-shell dual-channel emission materials remain largely unexplored^29,30^.

Compared to conventional closed-shell emitters, open-shell luminescent materials featuring unpaired electron have recently garnered considerable attention because of their unique excited-state properties. Diverse doublet emitters, such as organic radicals^31–35^, low-spin Fe(III) complexes^36–41^, and lanthanide Ce(III) complexes^42–46^, have been reported and investigated. Nevertheless, precise regulation of their excited-state relaxation dynamics, as well as the rational design of dual-channel emission pathways, are still in their infancy. To the best of our knowledge, only a few representative examples have been reported, with two organic radical molecules^47,48^ and a low-spin Fe(III) complex^49^. A deeper understanding of open-shell dual-channel emission is therefore essential for advancing their development and broadening their application in photonic and optoelectronic applications.

Among the aforementioned doublet emitters, Ce(III) complexes featuring 5d–4f emission have attracted considerable attention owing to their unique luminescent properties. These include tunable emission color arising from the ligand-field sensitivity of the 5d orbitals (in contrast to the shielded 4f orbitals) and short excited-state lifetimes on the nanosecond scale due to spin- and parity-allowed 5d–4f transitions^50,51^. As a lanthanide ion, Ce(III) exhibits diverse coordination environments, showing variable coordination numbers^52^. The variability stems from the shielding of the 4f orbitals, which results in weak ligand-field effects and predominantly electrostatic interactions between the lanthanide ions and the ligands^53^. Consequently, the small contribution of ligand-field stabilization energy allows for flexible and diverse coordination numbers. The rich coordination environment enables the photophysical properties of Ce(III) complexes to be well tuned; for example, the emission color of Ce(III) complexes covers a wide region from ultraviolet to almost near infrared^54^. However, many well-studied Ce(III) complexes typically show a single-channel emission originating from the lowest 5d orbital of the Ce(III) center according to Kasha’s rule, whereas dual-channel doublet emission based on Ce(III) ions remains underexplored.

**Fig. 1:**
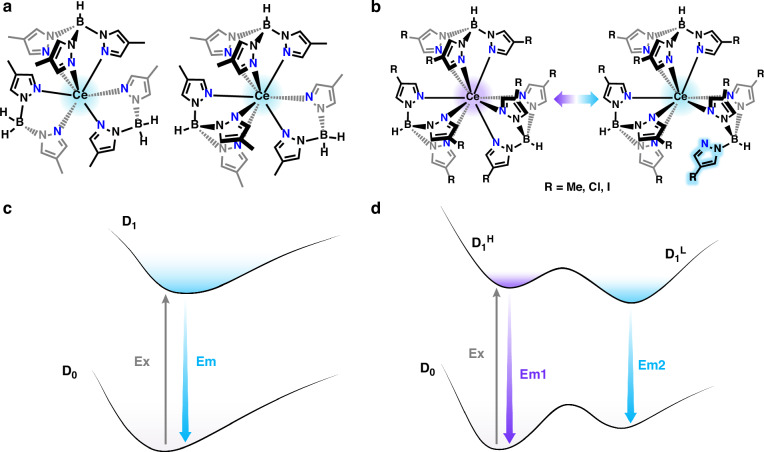
Molecular structure and energy diagram. **a** Molecular structures of Ce(Tp^4Me^)(Bp^4Me^)_2_ and Ce(Tp^4Me^)_2_(Bp^4Me^). **b** Molecular structures of Ce(Tp^4R^)_3_ (R = Me, Cl, I) with different coordination geometry. **c** The energy diagram of single-channel doublet emission. **d** The energy diagram of dual-channel doublet emission

Herein, we report dual-channel doublet emission in Ce(III) complexes Ce(Tp^4R^)_3_ (R = Me, Cl, I). To elucidate this phenomenon, reference complexes Ce(Tp^4Me^)(Bp^4Me^)_2_ and Ce(Tp^4Me^)_2_(Bp^4Me^) were designed and studied. Accompanied by density functional theory (DFT) calculations, the dual-channel doublet emission is attributed to the crowded and weak coordination interactions in Ce(Tp^4R^)_3_ (R = Me, Cl, I), leading to the variation and coexistence of nine- and eight-coordination geometries after high-energy light excitation (Fig. 1).

## Results

### Synthesis and structures

The synthetic routes for the ligands and their corresponding Ce(III) complexes are illustrated in **Scheme S1**. Pyrazole derivatives were purchased and used without further purification. KBp^4Me^ was obtained by heating 4-methylpyrazole and potassium borohydride (KBH_4_) until 2 equiv. of H_2_ had been evolved. KTp^4R^ (R = Me, Cl) were prepared by heating the corresponding pyrazole derivative and KBH_4_ until 3 equiv. of H_2_ had been evolved. KTp^4I^ was synthesized by refluxing 4-iodopyrazole (HPz^4I^) with KBH_4_ in xylene for 48 h. Ce(Tp^4Me^)(Bp^4Me^)_2_ was synthesized in a N_2_ glovebox by directly mixing KTp^4Me^ (1 equiv.), KBp^4Me^ (2 equiv.) and cerium triflate (Ce(OTf)_3_) (1 equiv.) in tetrahydrofuran (THF) and stirring overnight, followed by purification via thermal gradient sublimation under vacuum. The synthesis and purification of Ce(Tp^4Me^)_2_(Bp^4Me^) is similar to Ce(Tp^4Me^)(Bp^4Me^)_2_, but instead of a 2:1:1 molar ratio of KTp^4Me^, KBp^4Me^, and Ce(OTf)_3_ were used. Ce(Tp^4R^)_3_ (R = Me, Cl, I) were prepared in a N_2_ glovebox by adding the methanol solution of KTp^4R^ (3 equiv.) to the methanol solution of Ce(OTf)_3_ (1 equiv.) and stirring overnight. Ce(Tp^4Me^)_3_ and Ce(Tp^4Cl^)_3_ were purified by thermal gradient sublimation, while Ce(Tp^4I^)_3_ was purified by recrystallization from *n*-hexane/THF. The detailed experimental procedures for the synthesis of the ligands and corresponding Ce(III) complexes are provided in the Materials and Methods.

**Fig. 2:**
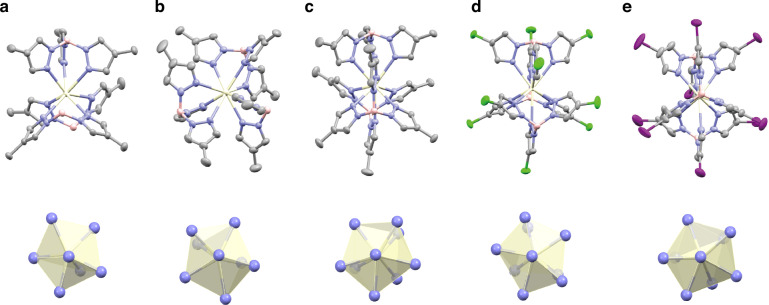
Crystal structures of Ce(III) complexes. **a** Ce(Tp^4Me^)(Bp^4Me^)_2_, (**b**) Ce(Tp^4Me^)_2_(Bp^4Me^), (**c**) Ce(Tp^4Me^)_3_, (**d**) Ce(Tp^4Cl^)_3_, (**e**) Ce(Tp^4I^)_3_. Coordination polyhedrons of the five Ce(III) complexes: Ce(Tp^4Me^)(Bp^4Me^)_2_, Ce(Tp^4Me^)_2_(Bp^4Me^), Ce(Tp^4Me^)_3_, Ce(Tp^4Cl^)_3_ and Ce(Tp^4I^)_3_ (Left to right). Ce atoms are represented in light-yellow, B in pink, N in blue, C in grey, Cl in green, and I in purple. H atoms are omitted for clarity

Single crystals of the five Ce(III) complexes were obtained by slow evaporation from a mixed solution of THF and *n*-hexane (Ce(Tp^4Me^)(Bp^4Me^)_2_ and Ce(Tp^4R^)_3_ (R = Me, Cl, I)) or thermal gradient sublimation (Ce(Tp^4Me^)_2_(Bp^4Me^)), and single-crystal X-ray diffraction (SXRD) was performed to investigate the coordinate geometry of these Ce(III) complexes. The crystal structures are presented in Fig. 2, and detailed crystal data are summarized in Tables S1 and S2. The bond lengths between Ce(III) and coordination N atoms are listed in Table 1. From Ce(Tp^4Me^)(Bp^4Me^)_2_ to Ce(Tp^4Me^)_2_(Bp^4Me^), then to Ce(Tp^4R^)_3_ (R= Me, Cl, I), the coordination number increases progressively from 7 to 8, then to 9, and the average bond length of Ce-N increases from 2.585 Å to 2.612 Å, then to ~2.67 Å. To evaluate the protection of the Ce(III) center afforded by the coordinating ligands, percent buried volume (%*V*_bur_) values of the Ce(III) complexes, which is defined as the fraction of the ligand volume relative to the total volume of a sphere centered on the metal, were calculated from their single-crystal structures. As shown in Figure. S1, with increasing coordination numbers, %*V*_bur_ values of these Ce(III) complexes also increase gradually from 89.3% to 92.0%, then to ~94%. Since the coordination between the Ce(III) center and the ligands involves weak electrostatic interactions, higher coordination numbers and more crowded coordination environments may lead to varied coordination geometries and diverse photophysical properties.

**Table 1:** The Ce-N bond lengths and average bond length of Ce(III) complexes

Bond (Å)	Ce(Tp^4Me^)(Bp^4Me^)_2_	Ce(Tp^4Me^)_2_(Bp^4Me^)	Ce(Tp^4Me^)_3_	Ce(Tp^4Cl^)_3_	Ce(Tp^4I^)_3_
Ce-N1	2.568	2.570	2.768	2.597	2.755
Ce-N2	2.579	2.655	2.619	2.631	2.629
Ce-N3	2.583	2.617	2.627	2.649	2.595
Ce-N4	2.590	2.563	2.768	2.779	2.755
Ce-N5	2.636	2.636	2.619	2.788	2.629
Ce-N6	2.568	2.594	2.627	2.597	2.595
Ce-N7	2.569	2.597	2.768	2.628	2.755
Ce-N8	—	2.664	2.619	2.758	2.629
Ce-N9	—	—	2.627	2.605	2.595
Average	2.585	2.612	2.671	2.670	2.660

### Photophysical properties

**Fig. 3:**
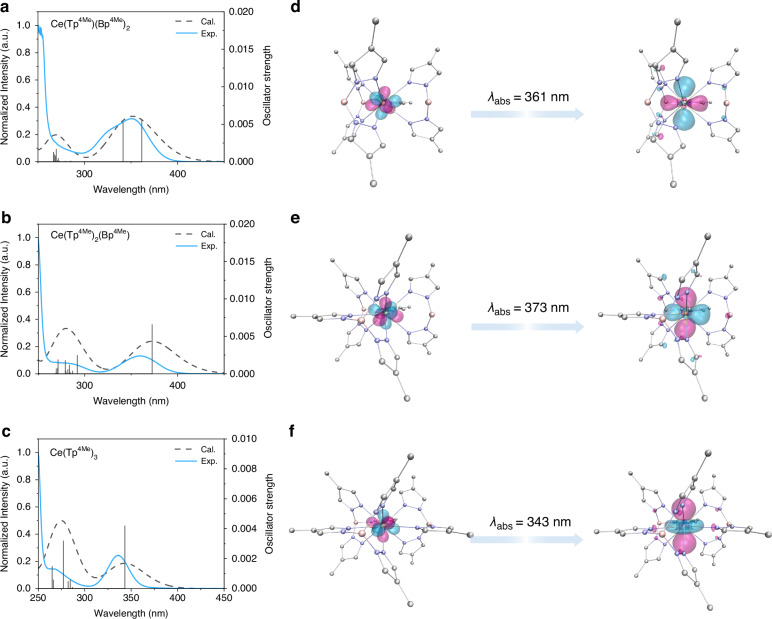
UV-vis absorption spectra of Ce(III) complexes in DCM solution (10^−3 ^M) and TD-DFT results calculated based on the ground-state optimized geometries. TD-DFT calculated (black dotted line) and experimental (blue solid line) absorption of (**a**) Ce(Tp^4Me^)(Bp^4Me^)_2_, (**b**) Ce(Tp^4Me^)_2_(Bp^4Me^), (**c**) Ce(Tp^4Me^)_3_. The predicted spectra were rendered as Gaussian line shapes. Oscillator strengths for the electronic transitions are shown as black vertical lines. Calculated NTOs of the lowest energy transition for (**d**) Ce(Tp^4Me^)(Bp^4Me^)_2_, (**e**) Ce(Tp^4Me^)_2_(Bp^4Me^), (**f**) Ce(Tp^4Me^)_3_ in the gas phase with an iso-surface value of ±0.05

The UV-vis absorption spectra of only Ce(Tp^4Me^)(Bp^4Me^)_2_, Ce(Tp^4Me^)_2_(Bp^4Me^) and Ce(Tp^4Me^)_3_ were measured in dichloromethane (DCM), since Ce(Tp^4R^)_3_ (R=Cl, I) are almost insoluble in DCM. Besides the absorption peaks below 260 nm that are mainly attributed to the ligand (Fig. 3a–c and Fig. S2, S3), the UV-vis spectra of Ce(Tp^4Me^)(Bp^4Me^)_2_, Ce(Tp^4Me^)_2_(Bp^4Me^) and Ce(Tp^4Me^)_3_ display absorption bands ranging from 260 nm to 400 nm with maximum molar extinction coefficients (*ε*_max_) of 716 L mol^−1^ cm^−^^1^, 746 L mol^−1^ cm^−1^ and 538 L mol^−1^ cm^−1^, respectively. These absorption bands are attributed to the parity-allowed 4f-5d transition of Ce(III), which is consistent with the time-dependent density functional theory (TD-DFT) results. According the natural transition orbitals (NTO) analysis (Fig. 3d–f), the lowest-energy absorption peaks occur at 361 nm, 373 nm and 343 nm for Ce(Tp^4Me^)(Bp^4Me^)_2_, Ce(Tp^4Me^)_2_(Bp^4Me^) and Ce(Tp^4Me^)_3_, respectively, corresponding to 4f_xyz_ to 5d_xz_/5d_yz_ transition in the first two complexes and 4f_xyz_ to 5d_z2_ transition in the latter complex.

**Fig. 4:**
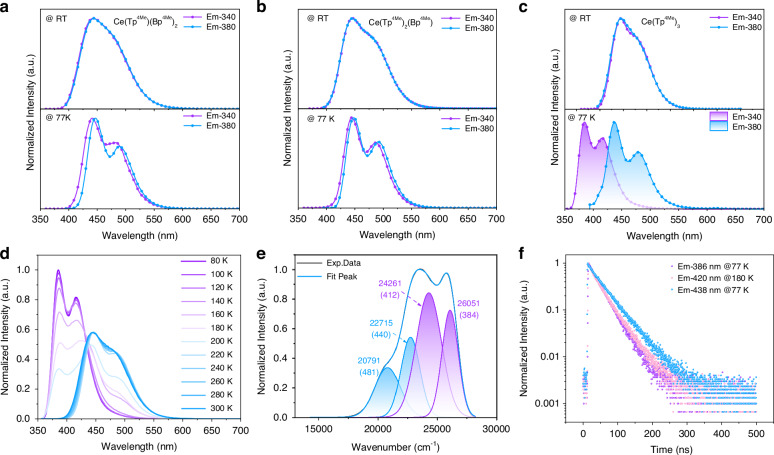
Photophysical properties of Ce(III) complexes in DCM solutions (10^−3 ^M). **a** Emission spectra of Ce(Tp^4Me^)(Bp^4Me^)_2_ at RT and 77 K with different excitation wavelengths. **b** Emission spectra of Ce(Tp^4Me^)_2_(Bp^4Me^) at RT and 77 K with different excitation wavelengths. **c** Emission spectra of Ce(Tp^4Me^)_3_ at RT and 77 K with different excitation wavelengths. **d** Temperature-dependent emission spectra of Ce(Tp^4Me^)_3_, the excitation wavelength is 340 nm. **e** Gaussian-peak fitting of the emission spectrum of Ce(Tp^4Me^)_3_ at 180 K. **f** Transient photoluminescence decays of Ce(Tp^4Me^)_3_. Emissions at 386 and 420 nm were measured with a 340 EPL source, and that at 438 nm with a 375 EPL source

The photoluminescence properties of Ce(Tp^4Me^)(Bp^4Me^)_2_, Ce(Tp^4Me^)_2_(Bp^4Me^) and Ce(Tp^4Me^)_3_ in DCM were measured both at room temperature (RT) and 77 K. At RT, the three complexes exhibit bright blue emissions under excitation wavelengths from 300 nm to 380 nm, with maximum emission peaks (*λ*_max_) located at 444 nm, 445 nm and 445 nm, respectively (Figs. 4a–c and Fig. S4). The full widths at half maxima (FWHMs) are 86 nm, 84 nm, and 83 nm for Ce(Tp^4Me^)(Bp^4Me^)_2_, Ce(Tp^4Me^)_2_(Bp^4Me^), and Ce(Tp^4Me^)_3_, respectively. To further elucidate the essence of the d-f transition, the PL spectra were analyzed by using Gaussian peak fitting. As shown in Fig. S5, each emission spectrum can be fitted into double peaks with an energy difference of about 2000 cm^−1^, corresponding to the transition from the 5d excited state to the two 4f ground states (^2^F_5/2_ and ^2^F_7/2_) in Ce(III) centers. As shown in Fig. S6, the excited-state lifetimes (*τ*) are 54 ns, 57 ns and 57 ns for Ce(Tp^4Me^)(Bp^4Me^)_2_, Ce(Tp^4Me^)_2_(Bp^4Me^) and Ce(Tp^4Me^)_3_, respectively, in agreement with reported values for Ce(III) complexes^43^. In addition, the photoluminescence quantum yields (PLQYs, *Φ*_PL_) of the three complexes were measured as 97%, 98% and 97%, respectively. To further evaluate the photophysical parameters of these Ce(III) complexes, the radiative rate constant (*k*_r_) and non-radiative rate constant (*k*_nr_) were calculated with the equation *k*_r_ = *Φ*_PL_/*τ* and *k*_nr_ = 1/*τ* - *k*_r_. As shown in Table 2, the values of *k*_r_ substantially exceed *k*_nr_, indicating the Ce(III) centers are effectively shielded by the surrounding ligands and the non-radiative decay pathways are suppressed efficiently.

**Table 2:** Photophysical data of Ce(III) complexes in DCM solution (10^−3 ^M) at room temperature

Ce(III) complexes	*λ*_em_^*a*^ [nm]	*Φ*_PL_^*a*^ [%]	*τ* [ns]	FWHM [nm]	*k*_r_ [10^7^ s^−1^]	*k*_nr_ [10^7^ s^−1^]
Ce(Tp^4Me^)(Bp^4Me^)_2_	444	97	54	86	1.8	0.056
Ce(Tp^4Me^)_2_(Bp^4Me^)	445	98	57	84	1.7	0.035
Ce(Tp^4Me^)_3_	445	97	57	83	1.7	0.053

^a^The excitation wavelength was 340 nm

At 77 K, Ce(Tp^4Me^)(Bp^4Me^)_2_ and Ce(Tp^4Me^)_2_(Bp^4Me^) show similar but more structured PL spectra compared to those at RT. The emission spectra of the two complexes split into two peaks located at 442 nm and 483 nm, 444 nm and 488 nm, respectively (Fig. 4a, b). The energy differences between these two peaks are approximately 2000 cm^−1^ (Figure. S7), further confirming that the emission originates from the 5d-4f transition. By varying the excitation wavelength from 300 nm to 380 nm, the PL spectra remain almost unchanged, indicating the emissions are excitation-independent (Figure. S8). In contrast, Ce(Tp^4Me^)₃ exhibits markedly different photoluminescent properties compared with Ce(Tp^4Me^)(Bp^4Me^)_2_ and Ce(Tp^4Me^)_2_(Bp^4Me^), as shown in Fig. 4c. Under 380 nm excitation, the emission spectrum is similar to that at RT, but with two obvious peaks at 438 nm and 480 nm. However, two new emission peaks emerge at 386 nm and 415 nm under 340 nm excitation, indicating the existence of a higher-lying 5d excited state. To gain insight into this unusual luminescence behavior, excitation spectra were recorded at the emission maxima of 438 nm and 386 nm (Fig. S9). In contrast to the excitation spectrum monitored at 386 nm, the excitation profile at 438 nm reveals the presence of an additional excitation band, indicating that the complex can emit from multiple excited states. As both the emissions are from the Ce(III) center, we tentatively ascribe the two emissions to two 5d excited states, denoted as low-energy D_1_^L^ and high-energy D_1_^H^.

Considering the distinct PL behavior of Ce(Tp^4Me^)_3_ at RT and 77 K, temperature-dependent emission spectra were performed (Fig. 4d) under 340 nm excitation. Starting from 300 K, the complex only shows a D_1_^L^ emission. With the temperature decreasing, the emission of D_1_^H^ emerges at 200 K. The complex exhibits dual-channel doublet emission from both D_1_^L^ and D_1_^H^ to D_0_ in the temperature range of 200–160 K. Further decrease in temperature results in the extinction of D_1_^L^ emission, and only the emission from the D_1_^H^ state can be observed. To further visualize the dual-channel doublet emission at 200–160 K, the emission spectrum of Ce(Tp^4Me^)_3_ at 180 K was fitted into four Gaussian peaks, for example, with wavenumbers of 20791 cm^−^^1^(481 nm), 22715 cm^−^^1^ (440 nm), 24261 cm^−1^ (412 nm) and 26051 cm^−1^ (384 nm), respectively (Fig. 4e). The former two peaks originate from the D_1_^L^ to the ground state D_0_ with an energy difference of 1924 cm^−1^, and the latter two peaks can be ascribed to the D_1_^H^ to the ground state D_0_ with an energy difference of 1790 cm^−1^.

To further understand the excited state dynamics of D_1_^L^ and D_1_^H^, the energy transfer rate (*k*_ET_) and radiative transition rate (*k*_r_) were calculated by using transient photoluminescence decays (Fig. 4f). Ce(Tp^4Me^)_3_ displays mono-exponential decay with *τ* of 45 ns at 438 nm (D_1_^L^ emission) and 33 ns at 386 nm (D_1_^H^ emission) at 77 K. Considering the *Φ*_PL_ of Ce(Tp^4Me^)_3_ is 97% (nearly 100%), thus the non-radiative transition rate is negligible. According to the equation that τ = 1/*k*_r_, the intrinsic radiative rates of D_1_^L^ and D_1_^H^ are approximately 2.2 × 10^7^ s^−^^1^ and 3.0 × 10^7^ s^−^^1^, respectively. At 180 K, the complex exhibits dual-channel doublet emission of D_1_^L^ and D_1_^H^ simultaneously with a bi-exponential decay of 43 ns and 21 ns at 420 nm, where 43 ns is assigned to the transition of D_1_^L^ → D_0_, while 21 ns encompasses the process of both the radiative decay of D_1_^H^ to D_0_ and energy transfer of D_1_^H^ to D_1_^L^. Therefore, the *k*_ET_ (D_1_^H^ → D_1_^L^) can be estimated as 1.7 × 10^7^ s^−1^ according to *τ* = 1/(*k*_r_ + *k*_ET_), which is slower than the radiative rate of D_1_^H^ → D_0_ (3.0 × 10^7^ s^−1^). Therefore, under high-energy excitation, Ce(Tp^4Me^)_3_ exhibits both the low-energy emission of D_1_^L^ → D_0_, the high-energy emission of D_1_^H^ → D_0_, and energy transfer from D_1_^H^ → D_1_^L^.

In addition, the photophysical properties of all five complexes in THF at RT and in 2-Methyltetrahydrofuran (2-MeTHF) at 77 K are closely similar to those observed in DCM, consistent with the Ce(III)-centered 5d-4f emission, which is only weakly affected by solvent polarity. The corresponding photophysical data are provided in Figs. S10–S12 and Tables S3, S4.

**Fig. 5:**
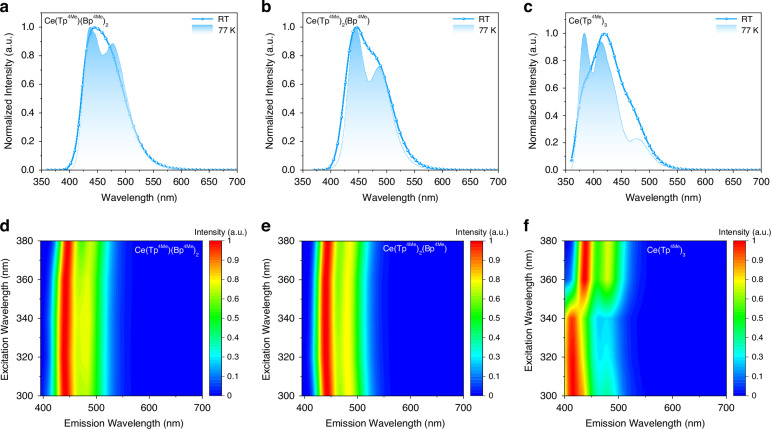
Photophysical properties of Ce(III) complexes in solid-powder state. **a** Emission spectra of Ce(Tp^4Me^)(Bp^4Me^)_2_. **b** Emission spectra of Ce(Tp^4Me^)_2_(Bp^4Me^). **c** Emission spectra of Ce(Tp^4Me^)_3_. **d** Excitation-emission mapping of Ce(Tp^4Me^)(Bp^4Me^)_2_ at 77 K. **e** Excitation-emission mapping of Ce(Tp^4Me^)_2_(Bp^4Me^) at 77 K. **f** Excitation-emission mapping of Ce(Tp^4Me^)_3_ at 77 K

Subsequently, the photophysical properties of Ce(Tp^4Me^)(Bp^4Me^)_2_, Ce(Tp^4Me^)_2_(Bp^4Me^) and Ce(Tp^4Me^)_3_ were investigated in solid-powder state. As shown in Fig. 5a, b, Ce(Tp^4Me^)(Bp^4Me^)_2_ and Ce(Tp^4Me^)_2_(Bp^4Me^) exhibit blue emissions at RT, with FWHM values of 81 nm and 83 nm, respectively. At 77 K, their PL spectra further split into two distinct peaks, which is a result of the ground 4f state of Ce(III) being divided into two sublevels. By using different excitation wavelengths ranging from 300 nm to 380 nm at 77 K (Fig. 5d, e), the emission spectra remain almost unchanged, indicating that the emission originates exclusively from the lowest doublet excited state, i.e., single-channel doublet emission similar to typically reported Ce(III) complexes. In contrast, Ce(Tp^4Me^)_3_ exhibits distinct photoluminescent properties, as shown in Fig. 5c. The complex displays three emission peaks at around 385 nm, 420 nm, and 465 nm, with a FWHM of 90 nm, differing from its spectrum in DCM at RT. At 77 K, a more well-defined spectrum with three distinct peaks at 384 nm, 414 nm, and 477 nm was observed. By varying the excitation wavelength from 300 nm to 380 nm in 20 nm steps (Fig. S13), the emission maxima shifted obviously (Fig. 5f). These results demonstrate that Ce(Tp^4Me^)₃ has multiple emissive 5d excited states and exhibits dual-channel doublet emission at both RT and 77 K in solid-powder state. Then, excited-state lifetimes of the three complexes were measured at RT. Ce(Tp^4Me^)(Bp^4Me^)_2_ and Ce(Tp^4Me^)_2_(Bp^4Me^) exhibit mono-exponential lifetimes of 43 ns and 49 ns, respectively (Fig. S14). While Ce(Tp^4Me^)_3_ exhibits dual-channel doublet emission (D_1_^L^ and D_1_^H^), leading to wavelength-dependent average excited-state lifetimes. The lifetimes increase from 31 ns at 385 nm to 38 ns at 420 nm and 44 ns at 465 nm (Fig. S15a), indicating that the progressive lengthening with red-shifted detection wavelengths reflects a longer excited-state lifetime for the D_1_^L^ excited state compared to the D_1_^H^ excited-state. All three complexes exhibit *Φ*_PL_ of ~100% in the solid-powder state, as a class of highly efficient blue emissive Ce(III) complexes.

**Fig. 6:**
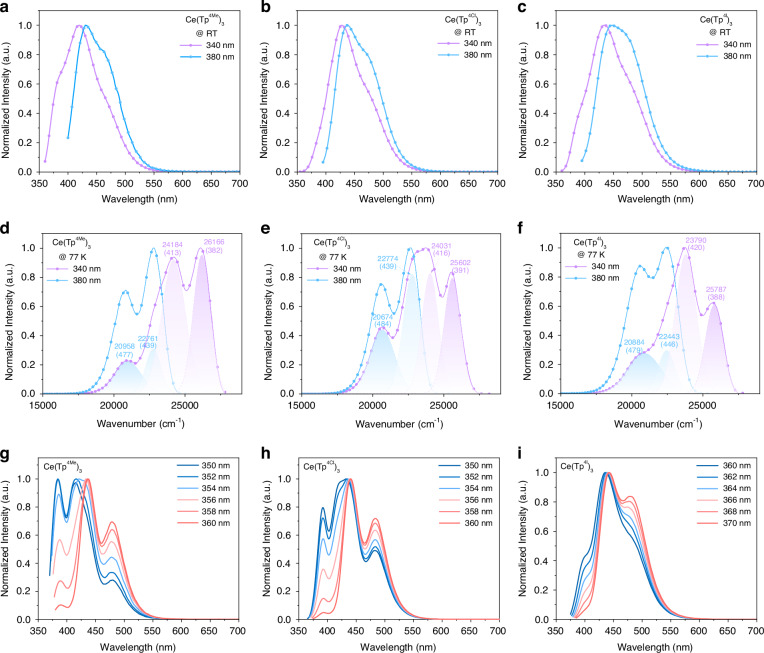
Photophysical properties of Ce(Tp^4R^)_3_ (R = Me, Cl, I) in solid-powder state. **a**–**c** Emission spectra of Ce(Tp^4Me^)_3_, Ce(Tp^4Cl^)_3_, Ce(Tp^4I^)_3_ at RT, respectively. **d**–**f** Gaussian-peak fitting of Ce(Tp^4Me^)_3_, Ce(Tp^4Cl^)_3_, Ce(Tp^4I^)_3_ at 77 K, respectively. **g**–**i** Excitation-dependent emission spectra of Ce(Tp^4Me^)_3_, Ce(Tp^4Cl^)_3_, Ce(Tp^4I^)_3_ at 77 K, respectively

Interestingly, when keeping the coordination environment of Ce(III) ion unchanged, while replacing the 4-position methyl with Cl or I atoms, the resulted Ce(III) complexes Ce(Tp^4R^)_3_ (R = Cl, I) showed similar photophysical properties to Ce(Tp^4Me^)_3_ in solid-powder state both at RT and 77 K (Fig. 6, Table 3). For example, all three complexes display dual-channel doublet emission under 340 nm excitation and show only low-energy emission at 380 nm excitation at RT (Fig. 6a–c). At 77 K, all three complexes exhibit a more structured spectrum which can be split into four Gaussian peaks (Fig. 6d–f), corresponding to emissions from D_1_^L^ and D_1_^H^. And all three complexes show excitation-dependent emission spectra (Fig. 6g–i) and wavelength-dependent excited-state lifetimes (Fig. S15). These results indicate that the dual-channel doublet emission in Ce(Tp^4R^)_3_ may arise from a nine-coordinated environment, as compared to Ce(Tp^4Me^)(Bp^4Me^)_2_ and Ce(Tp^4Me^)_2_(Bp^4Me^). The reproducible observation of dual-channel doublet emission in both purified solid-powder state and solution further indicates that this behavior is intrinsic to the Ce(III) complexes, rather than arising from sample purity. All five Ce(III) complexes show good thermal- (Fig. S16,S17), photo- (Fig. S18a), and air-stability (Fig. S18b), supporting their potential for practical applications.

**Table 3:** Photophysical data of Ce(III) complexes in solid-powder state at room temperature

Ce(III) complex	*λ*_max_^*a*^ [nm]	*Φ*_PL_^*b*^ [%]	*τ* [ns]	FWHM [nm]	*k*_r_ [10^7^ s^−^^1^]	*k*_nr_ [10^7^ s^−1^]
Ce(Tp^4Me^)(Bp^4Me^)_2_	445	~100	43	81	2.3	~0
Ce(Tp^4Me^)_2_(Bp^4Me^)	441	~100	49	83	2.0	~0
Ce(Tp^4Me^)_3_	420	~100	38	90	2.6	~0
Ce(Tp^4Cl^)_3_	428	~100	37	80	2.7	~0
Ce(Tp^4I^)_3_	435	41	22	93	1.9	2.7

^a^The excitation wavelength was 340 nm. ^***b***^The excitation wavelength was 320 nm for Ce(Tp^4Me^)_3_ and 340 nm for the other four complexes

### Computational investigations

To ascertain the origin of the dual-channel doublet emission in nine-coordinated Ce(Tp^4R^)_3_ (R = Me, Cl, I), the complexes Ce(Tp^4Me^)(Bp^4Me^)_2_, Ce(Tp^4Me^)_2_(Bp^4Me^), and Ce(Tp^4Me^)_3_ were selected as representatives for DFT and TD-DFT calculations. Detailed calculation methods were provided in the Materials and Methods.

The geometry optimization structures for the ground state (D_0_) and the first excited doublet state (D_1_) are shown in Fig. S19, and the optimized coordination bonds of the three Ce(III) complexes are listed in Table S5. Compared with the Ce-N bond lengths in the ground state, all Ce-N bond lengths in the excited-state have been shortened for Ce(Tp^4Me^)(Bp^4Me^)_2_ and Ce(Tp^4Me^)_2_(Bp^4Me^), suggesting that bonding interactions between the outer 5d orbital and the N atoms are stronger than those involving the inner 4f orbital. However, for Ce(Tp^4Me^)_3_, eight Ce-N bonds become shorter while one Ce-N bond shows a pronounced elongation in the excited state. This behavior may arise from excited-state structural relaxation, where the steric crowding in the nine-coordinate environment favors the formation of an eight-coordinate excited-state structure. Considering that one Ce-N bond elongates in the excited-state, the pyrazolyl fragment may rotate to a metastable conformation. To examine this possibility, the corresponding structure was constructed and subjected to geometry optimization (Fig. S20), and the optimized Ce-N bond lengths are summarized in Table S6. At the same computational level, the ground-state conformation with eight-coordination is 0.19 eV higher in energy than the nine-coordinated structure, suggesting the potential for a thermally activated conformational rearrangement^55^.

**Fig. 7:**
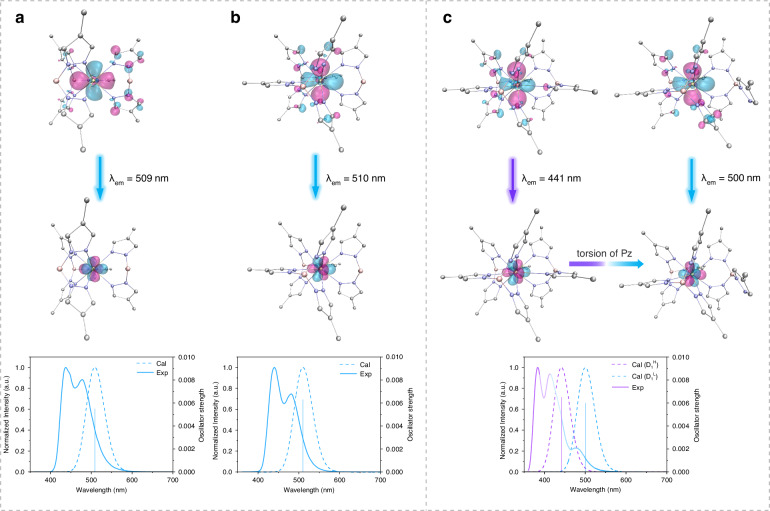
Emission characteristics of Ce(III) complexes by TD-DFT calculations. **a** The NTO analysis of Ce(Tp^4Me^)(Bp^4Me^)_2_ (top) and comparison between calculated and experimental emission spectra at 77 K in solid-powder state (bottom). **b** The NTO analysis of Ce(Tp^4Me^)_2_(Bp^4Me^) (top) and comparison between calculated and experimental emission spectra at 77 K in solid-powder state (bottom). **c** The NTO analysis of Ce(Tp^4Me^)_3_ with both nine-coordinated and eight-coordinated geometries (top) and comparison between calculated and experimental emission spectra at 77 K in solid-powder state (bottom). The predicted spectra were rendered as Gaussian line shapes. Oscillator strengths for the electronic transitions are shown as blue or purple vertical lines. Calculated NTOs in the gas phase with an iso-surface value of ±0.05

To further investigate the excited-state properties of these Ce(III) complexes, their emission spectra were calculated. Although the calculated emission maxima were underestimated by 0.35–0.40 eV relative to the experimental values, the overall spectral trends remained consistent, allowing for meaningful qualitative discussion. As illustrated in Fig. 7a, b, both Ce(Tp^4Me^)(Bp^4Me^)_2_ and Ce(Tp^4Me^)_2_(Bp^4Me^) exhibit single-channel doublet emission originating from the 5d_xz_/5d_yz_ to 4f_xyz_ transition with calculated emission wavelength at 509 nm (Exp: 437 nm) and 510 nm (Exp: 439 nm), respectively. In contrast, as shown in Fig. 7c, Ce(Tp^4Me^)_3_ displays dual-channel doublet emission due to the multiple excited states: a higher-energy emission (D_1_^H^) corresponding to the 5d_xz_/5d_yz_ to 4f_xyz_ transition, with a calculated emission at 441 nm (Exp: 384 nm), and a lower-energy emission (D_1_^L^) involving the same 5d_xz_/5d_yz_ to 4f_xyz_ transition but located at 500 nm (Exp: 439 nm). The calculated energy gap of 0.33 eV is in close agreement with the experimental values (~0.40 eV), further supporting the hypothesis for the existence of multiple radiative 5d excited states.

## Discussion

In summary, we report dual-channel doublet emission in luminescent lanthanide Ce(III) complexes. By rationally designing Ce(III) complexes with seven-, eight-, and nine-coordinated geometries, as well as systematically characterizing their photophysical properties, we show that highly efficient dual-channel emission requires both rigid multidentate ligands to suppress nonradiative decay and an adaptable coordination environment to access multiple emissive excited states. Accordingly, the seven-coordinated Ce(Tp^4Me^)(Bp^4Me^)_2_ and the eight-coordinated Ce(Tp^4Me^)_2_(Bp^4Me^) showing rigid molecular structure while fixed coordination number exhibited classic single-channel doublet emission originating from the lowest 5d excited state with blue emission and high PLQY around 100%, while the nine-coordinated Ce(Tp^4R^)_3_ (R = Me, Cl, I) having rigid molecular structure and tunable coordination number showed dual-channel doublet emission both in DCM solution at low temperature and in solid-powder state even at room temperature, with blue emission and the highest PLQY up to ~100%. Crystal structure studies and theoretical calculations reveal that the crowded coordination environments and longer coordination bonds in the nine-coordinated complexes play a crucial role, which prefer to undergo a structural relaxation from nine-coordination to eight-coordination after high-energy light excitation, resulting in two different coordination environments for Ce(III) and hence dual-channel emission. This study not only unveils an intriguing luminescent phenomenon in Ce(III) complexes but also provides new insights into the design of advanced dual-channel emission materials with unique photophysical properties.

## Materials and Methods

### General information

All chemical reagents used in the synthesis were commercially available and were used as received unless otherwise mentioned. Ce(III) complexes were synthesized and purified in a glove box with nitrogen atmosphere. THF, 2-MeTHF and *n*-hexane were distilled using Na, NaH, and benzophenone under a nitrogen atmosphere. Dichloromethane (DCM) was distilled using CaH_2_ under a nitrogen atmosphere. ^1^H NMR spectra were recorded on a Bruker-400 MHz NMR spectrometer using tetramethylsilane (TMS) as an internal reference. Elemental analysis of complexes was tested by a VARIO EL analyzer (GmbH, Hanau, Germany).

### Percent buried volume calculations

The percent buried volume (%*V*_bur_)^56^ values of Ce(III) complexes were calculated using the SambVca web application, with the XYZ coordinates of each complex used as the input structure. Hydrogen atoms were included in the calculations. The calculations were performed using Bondi radii scaled by 1.17, a sphere radius of 3.5 Å, and a mesh spacing of *s* = 0.10 Å.

### Synthesis

#### Synthesis of KBp^4Me^

Under a N_2_ atmosphere, 4-methyl-1*H*-pyrazole (HPz^4Me^) (8.223 g, 100.2 mmol) and KBH_4_ (2.171 g, 40.25 mmol) were added in a 100 mL round-bottom flask and heated at 120 °C until 2 equivalents of H_2_ were released. The crude product was stirred with toluene at 80 °C for 1 h and filtered to obtain a white solid. Yield: 48% (4.158 g). ^1^H NMR (400 MHz, DMSO-*d*_6_): δ 7.05 (t, *J* = 0.9 Hz, 1H), 6.97 (s, 1H), 1.92 (s, 3H).

#### Synthesis of KTp^4Me^

Under a N_2_ atmosphere, HPz^4Me^ (11.737 g, 143.0 mmol) and KBH_4_ (1.924 g, 35.67 mmol) were added in a 100 mL round-bottom flask and heated until 3 equivalents of H_2_ were released. The crude product was stirred with toluene at 80 °C for 1 h and filtered to obtain a white solid. Yield: 75% (7.894 g). ^1^H NMR (400 MHz, DMSO-*d*_6_): δ 7.14 (s, 1H), 6.95 (s, 1H), 1.94 (s, 3H).

#### Synthesis of KTp^4Cl^

Under a N_2_ atmosphere, 4-chloro-1*H*-pyrazole (HPz^4Cl^) (3.085 g, 30.09 mmol) and KBH_4_ (0.323 g, 5.99 mmol) were added in a 100 mL round-bottom flask and heated until 3 equivalents of H_2_ were released. The crude product was stirred with toluene at 80 °C for 1 h and filtered to obtain a white solid. Yield: 61% (1.302 g). ^1^H NMR (400 MHz, DMSO-*d*_6_) δ 7.42 (d, *J* = 0.7 Hz, 1H), 7.40 (d, *J* = 0.6 Hz, 1H).

#### Synthesis of KTp^4I^

Under a N_2_ atmosphere, 4-iodo-1*H*-pyrazole (HPz^4I^) (4.267 g, 22.00 mmol), KBH_4_ (0.539 g, 9.99 mmol) and 50 mL xylene were added in a 100 mL round-bottom flask and the mixture was refluxed for 48 h. The crude product was filtered and washed with toluene to obtain a white solid. Yield: 62% (3.900 g). ^1^H NMR (400 MHz, DMSO-*d*_6_): δ 7.42 (d, *J* = 3.1 Hz, 2H).

#### Synthesis of Ce(Tp^4Me^)(Bp^4Me^)_2_

In a glovebox with N_2_ atmosphere, KTp^4Me^ (0.593 g, 2.02 mmol) and KBp^4Me^ (0.862 g, 4.03 mmol) were dissolved in 30 mL THF. Then a suspension of Ce(OTf)_3_ (1.171 g, 1.99 mmol) in 20 mL THF was added dropwise to the solution. The mixture was stirred at room temperature overnight, and then the solvent was removed under vacuum. The resulting solid was sublimed under 10^−^^4^ Pa with a gradient temperature of 210°C–120°C–60°C to obtain a white solid. Yield: 20% (0.300 g). Elemental analysis Calcd (%) for C_28_H_40_B_3_CeN_14_: C 45.13, H 5.41, N 26.31; Found: C 45.43, H 5.48, N 26.66.

#### Synthesis of Ce(Tp^4Me^)_2_(Bp^4Me^)

The preparation was similar to Ce(Tp^4Me^)(Bp^4Me^)_2_ but instead of KTp^4Me^ (0.588 g, 2.00 mmol), KBp^4Me^ (0.214 g, 1.00 mmol) and Ce(OTf)_3_ (0.587 g, 1.00 mmol) were used and the resulting solid was sublimed with a gradient temperature of 210°C–120°C–60°C to obtain a white solid. Yield: 37% (0.305 g). Elemental analysis Calcd (%) for C_32_H_44_B_3_CeN_16_: C 46.58, H 5.37, N 27.15; Found: C 46.20, H 5.13, N 27.28.

#### Synthesis of Ce(Tp^4Me^)_3_

Under a N_2_ atmosphere, KTp^4Me^ (0.971 g, 3.30 mmol) was dissolved in 20 mL ultra-dry methanol. Then the solution of Ce(OTf)_3_ (0.587 g, 1.00 mmol) in 10 mL ultra-dry methanol was added dropwise to the solution. The mixture was stirred at room temperature overnight and filtered to obtain the crude product. The crude product was sublimed under 10^−^^4^ Pa with a gradient temperature of 270°C–200°C–50°C to obtain a white solid. Yield: 82% (0.742 g). Elemental analysis Calcd (%) for C_36_H_48_B_3_CeN_18_: C 47.75, H 5.34, N 27.84; Found: C 47.91, H 5.144, N 28.20.

#### Synthesis of Ce(Tp^4Cl^)_3_

The synthesis was similar to Ce(Tp^4Me^)_3_ but instead KTp^4Cl^ (1.632 g, 4.59 mmol) and Ce(OTf)_3_ (0.818 g, 1.39 mmol) were used and the resulting solid was sublimed with a gradient temperature of 280°C–160°C–60°C to obtain a white solid. Yield: 68% (1.023 g). Elemental analysis Calcd (%) for C_27_H_21_B_3_CeCl_9_N_18_: C 29.77, H 1.94, N 23.15; Found: C 29.77, H 1.91, N 23.31.

#### Synthesis of Ce(Tp^4I^)_3_

The synthesis was similar to that of Ce(Tp^4Me^)_3_ but instead KTp^4I^ (0.939 g, 1.49 mmol) and Ce(OTf)_3_ (0.274 g, 0.466 mmol) were used. The crude product was recrystallized in *n*-hexane and THF to obtain a white solid. Yield: 46% (0.408 g). Calcd (%) for C_27_H_21_B_3_CeI_9_N_18_·3C_6_H_14_: C 24.90, H 2.93, N 11.61; Found: C 24.75, H 2.46, N 11.22.

### Single-crystal X-ray crystallography

Single-crystal X-ray diffraction (SXRD) data were collected on a Rigaku Mercury CCD diffractometer utilizing the Mo Kα emission line (λ = 0.71069 Å). Relevant SXRD data were collected using the CrystalClear software.

### Spectral measurements

The photophysical properties of all complexes were measured under a nitrogen atmosphere unless otherwise specified. Steady-state and transient photoluminescence spectra were measured by an Edinburgh Analytical Instruments FLS980 spectrophotometer with an Xe lamp or pulsed lasers. Temperature-dependent steady-state spectra were recorded on the same Edinburgh Analytical Instruments FLS980 spectrophotometer using an Oxford temperature control accessory to adjust the temperature from 80 to 300 K. Photoluminescent quantum yield (PLQY) was acquired on a Hamamatsu C9920-02 PLQY measurement system with an integrating sphere.

### Computational details

All calculations were performed with the Gaussian 16, Rev A.03 package. Geometry optimization started from the single crystal structures. The ground state geometries were optimized using PBE0 hybrid density functional^57,58^ with dispersion correction (“D3BJ”). A 47-electron large-core pseudopotential MWB47 basis was employed for Ce(III) and the 6–31 G* basis set^59–61^ for all other atoms. Time-dependent DFT (TD-DFT) calculations, excited-state optimizations, and the single point calculations were carried out at the same computational level, except that Ce(III) was described with a 28-electron small-core pseudopotential MWB28 basis^62^. Natural transition orbitals (NTO) analysis was conducted using the Multiwfn program^63^, and rendered with the program visual molecular dynamics (VMD)^64^.

## Supplementary information

Supplementary Information

Ce(Tp4Me)(Bp4Me)2 cif

Ce(Tp4Me)2(Bp4Me) cif

Ce(Tp4Me)3 cif

Ce(Tp4Cl)3 cif

Ce(Tp4I)3 cif

## Data Availability

Crystallography data have been deposited at the Cambridge Crystallographic Data Centre (CCDC) under deposition numbers CCDC 2497163 (Ce(Tp^4Me^)(Bp^4Me^)_2_), 2497162 (Ce(Tp^4Me^)_2_(Bp^4Me^)), 2031983 (Ce(Tp^4Me^)_3_)^65^, 2497164 (Ce(Tp^4Cl^)_3_), and 2497165 (Ce(Tp^4I^)_3_), and are publicly available as of the date of publication.
