# Supplementary material for: Highly efficient dual-channel doublet emission in lanthanide cerium(III) complex
Source: Light Sci Appl. 2026 Aug 7;15:342. doi: 10.1038/s41377-026-02377-4 (PMC13451352; doi:10.1038/s41377-026-02377-4)
Supplement: Supplementary file 1 [file 41377_2026_2377_MOESM1_ESM.docx]

# **Supplementary Information**

**Highly Efficient Dual-Channel Doublet Emission in Lanthanide Cerium(III) Complex**

Yujia Li^#^, Peiyu Fang^#^, Zeyou Xiong, Jiayin Zheng, Huanyu Liu, Rubing Bai, Zuqiang Bian, Zhiwei Liu*

Beijing National Laboratory for Molecular Sciences, College of Chemistry and Molecular Engineering, Peking University, Beijing 100871, China

#These authors contribute equally to this work

*Email: zwliu@pku.edu.cn

**Scheme S1.** The synthesis route of ligands and corresponding Ce(III) complexes.

**Table S1**. Crystallographic data for Ce(Tp^4Me^)(Bp^4Me^)_2_ and Ce(Tp^4Me^)_2_(Bp^4Me^).

| Compound | Ce(Tp^4Me^)(Bp^4Me^)_2_ | Ce(Tp^4Me^)_2_(Bp^4Me^) |
| --- | --- | --- |
| chemical formula | C_28_H_40_B_3_CeN_14_ | C_32_H_44_B_3_CeN_16_ |
| formula weight | 745.29 | 825.38 |
| crystal size (mm) | 0.02×0.02×0.02 | 0.02×0.02×0.01 |
| temperature (K) | 180(10) | 180.00(10) |
| radiation | 0.71073 | 0.71073 |
| crystal system | Triclinic | monoclinic |
| space group | *P*$\bar{1}$ | *P*2_1_/*n* |
| *a*(Å) | 10.6905(2) | 19.3375(3) |
| *b*(Å) | 10.8704(2) | 19.7676(3) |
| *c*(Å) | 16.7913(4) | 21.6981(4) |
| *α*(°) | 84.3513(18) | 90.0 |
| *β*(°) | 82.0910(18) | 108.5078(18) |
| *γ*(°) | 65.694(2) | 90.0 |
| *V*(Å^3^) | 1759.55(7) | 7865.3(2) |
| Z | 2 | 4 |
| *ρ*(_calc_) (g/cm^3^) | 1.407 | 1.394 |
| F (000) | 758 | 3368 |
| absorp. coeff. (mm^-1^) | 1.335 | 1.203 |
| *θ* range (deg) | 2.102–29.384 | 1.980–29.479 |
| reflns collected | 10330  (R_int_ = 0.0389) | 24332  (R_int_ = 0.0333) |
| indep. reflns | 24308 | 61022 |
| refns obs. [*I*＞2*σ(I)*] | 7786 | 15401 |
| data/restr/paras | 8827/438/0 | 19805/969/0 |
| GOF | 1.033 | 1.086 |
| R_1_/wR_2_ [*I*＞2*σ(I)*] | 0.0280/0.0617 | 0.0364/0.0807 |
| R_1_/wR_2_ (all data) | 0.0357/0.0636 | 0.0525/0.0857 |

**Table S2**. Crystallographic data for Ce(Tp^4Me^)_3_ Ce(Tp^4Cl^)_3_ and Ce(Tp^4I^)_3_.

| Compound | Ce(Tp^4Me^)_3_ | Ce(Tp^4Cl^)_3_ | Ce(Tp^4I^)_3_ |
| --- | --- | --- | --- |
| chemical formula | C_36_H_48_B_3_CN_18_ | C_27_H_21_B_3_CeCl_9_N_18_C_4_H_8_O | C_27_H_21_B_3_CeI_9_N_18_ |
| formula weight | 905.47 | 1161.32 | 1912.28 |
| crystal size (mm) | 0.4×0.08×0.06 | 0.04×0.03×0.02 | 0.02×0.02×0.01 |
| temperature (K) | 180.00(10) | 180.00(10) | 179.99(10) |
| radiation | 0.71073 | 0.71073 | 0.71073 |
| crystal system | trigonal | monoclinic | cubic |
| space group | *R*$\bar{3}$*c* | *P*2_1_/*n* | *Pa*$\bar{3}$ |
| *a*(Å) | 21.0177(2) | 11.7531(2) | 24.3469(1) |
| *b*(Å) | 21.0177(2) | 18.8308(3) | 24.3469(1) |
| *c*(Å) | 33.7010(5) | 21.4891(4) | 24.3469(1) |
| *α*(°) | 90 | 90.0 | 90 |
| *β*(°) | 90 | 104.2483(18) | 90 |
| *γ*(°) | 120 | 90.0 | 90 |
| *V*(Å^3^) | 12892.7(3) | 4609.69(14) | 14432.16(11) |
| Z | 12 | 4 | 8 |
| *ρ*(_calc_) (g/cm^3^) | 1.399 | 1.673 | 1.761 |
| F (000) | 5556 | 2300 | 6848.455 |
| absorp. coeff. (mm^-1^) | 1.109 | 1.560 | 4.513 |
| *θ* range (deg) | 2.654–27.482 | 1.956–30.711 | 1.67–30.73 |
| reflns collected | 12205  (R_int_ = 0.0168) | 23188  **(**R_int_ = 0.0381**)** | 43367  **(**R_int_ = 0.0204**)** |
| indep. reflns | 15228 | 37849 | 148942 |
| refns obs. [*I*＞2*σ(I)*] | 2772 | 9904 | 4987 |
| data/restr/paras | 1305/86/1 | 12198/578/2 | 7095/175/0 |
| GOF | 1.053 | 1.036 | 1.1945 |
| R_1_/wR_2_ [*I*＞2*σ(I)*] | 0.0215/0.0590 | 0.0311/0.0788 | 0.0527/0.1471 |
| R_1_/wR_2_ (all data) | 0.0175/0.0447 | 0.0428/0.0838 | 0.0755/ 0.1847 |

**Fig. S1.** Buried volume *%V*_bur_ calculated for Ce(III) complexes. **a** Ce(Tp^4Me^)(Bp^4Me^)_2_, **b** Ce(Tp^4Me^)_2_(Bp^4Me^), **c** Ce(Tp^4Me^)_3_, **d** Ce(Tp^4Cl^)_3_ and **e** Ce(Tp^4I^)_3_.

**Fig. S2.** UV-vis absorption spectra of Ce(Tp^4Me^)(Bp^4Me^)_2_, Ce(Tp^4Me^)_2_(Bp^4Me^) and Ce(Tp^4Me^)_3_ in DCM solution.

**Fig. S3** Time-dependent UV-vis absorption spectra of Ce(III) complexes in DCM (10^-3^ M under a N_2_ atmosphere). **a** Ce(Tp^4Me^)(Bp^4Me^)_2_; **b** Ce(Tp^4Me^)_2_(Bp^4Me^); **c** Ce(Tp^4Me^)_3_.

**Fig. S4.** Excitation-emission mapping of Ce(III) complexes in DCM solution (10^-3^ M) at room temperature. **a** Ce(Tp^4Me^)(Bp^4Me^)_2_, **b** Ce(Tp^4Me^)_2_(Bp^4Me^) and **c** Ce(Tp^4Me^)_3_.

**Fig. S5.** Gaussian-peak fitting of Ce(III) complexes in DCM solution (10^-3^ M) at room temperature: **a** Ce(Tp^4Me^)(Bp^4Me^)_2_, **b** Ce(Tp^4Me^)_2_(Bp^4Me^) and **c** Ce(Tp^4Me^)_3_, the excitation wavelength is 340 nm.

**Fig. S6.** Transient PL decays in DCM solution (10^-3^ M) at room temperature for **a** Ce(Tp^4Me^)(Bp^4Me^)_2_, **b** Ce(Tp^4Me^)_2_(Bp^4Me^) and **c** Ce(Tp^4Me^)_3_.

**Fig. S7.** Gaussian-peak fitting for **a** Ce(Tp^4Me^)(Bp^4Me^)_2_ and **b** Ce(Tp^4Me^)_2_(Bp^4Me^) in DCM solution (10^-3^ M) at 77K. The excitation wavelength is 340 nm.

**Fig. S8.** PL spectra with different excitation wavelengths of **a** Ce(Tp^4Me^)(Bp^4Me^)_2_ and **b** Ce(Tp^4Me^)_2_(Bp^4Me^) in DCM solution (10^-3^ M) at 77K. Excitation-emission mapping of **c** Ce(Tp^4Me^)(Bp^4Me^)_2_ and **d** Ce(Tp^4Me^)_2_(Bp^4Me^) in DCM solution (10^-3^ M) at 77K.

**Fig. S9.** Excitation spectra of Ce(Tp^4Me^)_3_ in DCM solution (10^-3^ M) at 77 K, monitored at different emission wavelengths.

**Fig. S10.** Time-dependent UV-vis absorption spectra of Ce(III) complexes in THF (10^-3^ M under a N_2_ atmosphere). **a** Ce(Tp^4Me^)(Bp^4Me^)_2_; **b** Ce(Tp^4Me^)_2_(Bp^4Me^); **c** Ce(Tp^4Me^)_3_; **d** Ce(Tp^4Cl^)_3_; **e** Ce(Tp^4I^)_3_.

**Fig. S11.** Emission spectra of Ce(III) complexes in THF (RT) and 2-MeTHF (77 K) (10^-3^ M under a N_2_ atmosphere). **a** Ce(Tp^4Me^)(Bp^4Me^)_2_; **b** Ce(Tp^4Me^)_2_(Bp^4Me^); **c** Ce(Tp^4Me^)_3_; **d** Ce(Tp^4Cl^)_3_; **e** Ce(Tp^4I^)_3_.

**Fig. S12.** Transient photoluminescence decays of Ce(III) complexes. **a** RT with 340 EPL as excitation source in THF. **b** 77 K with 340 EPL as excitation source in 2-MeTHF. **c** 77 K with 375 EPL as excitation source in 2-MeTHF.

**Table S3.** Photophysical data of Ce(III) complexes in THF at RT.

| Ce(III) complex | *λ*_max_***^a^***  [nm] | *Φ*_PL_***^b^***  [%] | *τ*  [ns] | FWHM  [nm] | *k*_r_  [10^7^ s^-1^] | *k*_nr_  [10^7^ s^-1^] |
| --- | --- | --- | --- | --- | --- | --- |
| Ce(Tp^4Me^)(Bp^4Me^)_2_ | 430 | 89 | 52 | 83 | 1.2 | 0.21 |
| Ce(Tp^4Me^)_2_(Bp^4Me^) | 445 | 90 | 59 | 85 | 1.5 | 0.17 |
| Ce(Tp^4Me^)_3_ | 446 | 100 | 59 | 85 | 1.7 | 0 |
| Ce(Tp^4Cl^)_3_ | 442 | 98 | 55 | 83 | 1.8 | 0.04 |
| Ce(Tp^4I^)_3_ | 445 | 80 | 50 | 85 | 1.6 | 0.40 |

***^a^***The excitation wavelength was 340 nm. ***^b^***The excitation wavelength was 340 nm.

**Table S4.** Photophysical data of Ce(III) complexes in 2-MeTHF at 77 K.

| Ce(III) complex | *λ*_max_***^a^***  [nm] | *τ****^a^***  [ns] | FWHM***^a^***  [nm] | *λ*_max_^b^  [nm] | *τ*^b^  [ns] | FWHM^b^  [nm] |
| --- | --- | --- | --- | --- | --- | --- |
| Ce(Tp^4Me^)(Bp^4Me^)_2_ | 419 | 40 | 71 | 427 | 42 | 88 |
| Ce(Tp^4Me^)_2_(Bp^4Me^) | 445 | 45 | 76 | 449 | 47 | 80 |
| Ce(Tp^4Me^)_3_ | 385 | 34 | 57 | 447 | 47 | 79 |
| Ce(Tp^4Cl^)_3_ | 381 | 32 | 57 | 442 | 36 | 80 |
| Ce(Tp^4I^)_3_ | 382 | 29 | 57 | 445 | 38 | 80 |

***^a^***D_1_^H^ emission: The excitation wavelength was 340 nm for emission spectra; The excitation source was 340 EPL for transient photoluminescence decays. ***^b^*** D_1_^L^ emission: The excitation wavelength was 380 nm for emission spectra; The excitation source was 375 EPL for transient photoluminescence decays.

**Fig. S13.** The excitation spectra of Ce(Tp^4Me^)_3_ in solid-powder state at 77 K, monitored at different emission wavelengths.

**Fig. S14.** Transient PL decays in solid-powder state at room temperature of **a** Ce(Tp^4Me^)(Bp^4Me^)_2_ and **b** Ce(Tp^4Me^)_2_(Bp^4Me^).

**Fig. S15.** Transient PL decays in solid-powder state at room temperature of **a** Ce(Tp^4Me^)_3_, **b** Ce(Tp^4Cl^)_3_ and **c** Ce(Tp^4I^)_3_.

**Fig. S16.** Thermalgravimetric analysis (TGA) curves of Ce(III) complexes. *T*_d_ is defined as the temperature at 5% weight loss. **a** Ce(Tp^4Me^)(Bp^4Me^)_2_. **b** Ce(Tp^4Me^)_2_(Bp^4Me^). **c** Ce(Tp^4Me^)_3_. **d** Ce(Tp^4Cl^)_3_. **e** Ce(Tp^4I^)_3_.

**Fig. S17.** Integrated emission intensities of Ce(III) complexes at different temperatures.

**Fig. S18.** Time-dependent PLQYs of the Ce(III) complexes. **a** PLQY changes of 1 wt% doped PMMA films under continuous 365 nm irradiation (500 mW cm^-2^). **b** PLQY changes of Ce(III) complexes in the solid-powder state in air.

**Fig. S19.** Calculated geometry structures of **a** Ce(Tp^4Me^)(Bp^4Me^)_2_, **b** Ce(Tp^4Me^)_2_(Bp^4Me^) and **c** Ce(Tp^4Me^)_3_ in the ground state. Calculated geometries of **d** Ce(Tp^4Me^)(Bp^4Me^)_2_, **e** Ce(Tp^4Me^)_2_(Bp^4Me^) and **f** Ce(Tp^4Me^)_3_ in the D_1_ excited state.

**Table S5.** Optimized coordination bond lengths of Ce(III) complexes in the ground state (gs) and the D_1_ excited state (es).

|  | Ce(Tp^4Me^)(Bp^4Me^)_2_ | | Ce(Tp^4Me^)_2_(Bp^4Me^) | | Ce(Tp^4Me^)_3_ | |
| --- | --- | --- | --- | --- | --- | --- |
|  | gs | es | gs | es | gs | es |
| *r*_Ce-N_  (Å) | 2.5711 | 2.4948 | 2.6039 | 2.5263 | 2.6105 | 2.5222 |
|  | 2.5688 | 2.4941 | 2.6058 | 2.5262 | 2.6109 | 2.5221 |
|  | 2.6021 | 2.4963 | 2.6394 | 2.5486 | 2.7361 | 2.6493 |
|  | 2.5614 | 2.4117 | 2.5862 | 2.4558 | 2.6103 | 2.4488 |
|  | 2.5659 | 2.4126 | 2.5864 | 2.4559 | 2.6106 | 2.4488 |
|  | 2.5485 | 2.4529 | 2.6605 | 2.5754 | 2.7366 | 2.6616 |
|  | 2.5507 | 2.4524 | 2.5789 | 2.4718 | 2.6102 | 2.5228 |
|  | — | — | 2.5742 | 2.4717 | 2.6106 | 2.5226 |
|  | — | — | — | — | 2.7361 | 3.1167 |

**Fig. S20.** Calculated geometry structures of **a** Ce(Tp^4Me^)_3_ in the ground state with eight-coordination and **b** Ce(Tp^4Me^)_3_ in the D_1_ excited state with eight-coordination.

**Table S6.** Optimized coordination bond lengths of Ce(Tp^4Me^)_3_ in the ground state (gs) and the D_1_ excited state (es) with eight-coordination.

|  | Ce(Tp^4Me^)_3_-  [eight coordination] | |
| --- | --- | --- |
|  | gs | es |
| *r*_Ce-N_  (Å) | 2.654 | 2.572 |
|  | 2.582 | 2.464 |
|  | 2.593 | 2.465 |
|  | 2.653 | 2.539 |
|  | 2.616 | 2.502 |
|  | 2.561 | 2.559 |
|  | 2.579 | 2.485 |
|  | 2.586 | 2.495 |
